# Oxygen isotopes in ophicalcites: an ever-lasting controversy?

**DOI:** 10.1007/s00531-020-01934-5

**Published:** 2020-10-09

**Authors:** Daniel Bernoulli, Helmut Weissert

**Affiliations:** Department of Earth Sciences, Swiss Federal Institute of Technology (ETH), 8092 Zürich, Switzerland

**Keywords:** Ophicalcite, Oxygen-isotopes, Ocean–continent transition, Jurassic, Alps, Graubünden

## Abstract

Tectono-sedimentary breccias, known as ophicalcites, overlie serpentinised peridotites at a Jurassic ocean–continent transition along the Penninic-Austroalpine transition in the Eastern Alps of Switzerland. Deformation of the exhumed mantle rocks and breccia formation occurred under decreasing temperatures and along low-angle detachment faults exposing the mantle rocks at the sea floor and was coupled with hydrothermal activity and carbonation of the serpentinites at shallow depth and/or at the sea floor. Carbon isotopes in the ophicalcites persistently show marine values; however, the interpretation of oxygen-isotope values remained controversial: are they related to Jurassic hydrothermal activity or do they reflect Alpine metamorphic overprint? Here we discuss recent interpretations that relate oxygen isotope values measured in ophicalcites exclusively to Jurassic hydrothermal activity; to this end we use data that we earlier obtained along a north–south profile across Graubünden (eastern Switzerland). We revisited the sites of controversial interpretation along a north–south profile in eastern Switzerland. Along this profile, oxygen isotope values in ophicalcites and overlying pelagic sediments, up to 25 my younger than the ophicalcites, show identical values and become systematically lower with increasing Alpine metamorphism; they strongly deviate from values in ophicalcites and pelagic sediments measured along the Mid-Atlantic Ridge or ancient Atlantic ocean-continent transitions as e.g. in the Iberia–Newfoundland transect. The oxygen-isotope values measured in Alpine ophicarbonates thus reflect isotopic resetting during the Alpine orogeny, related to fluid-rock interaction during regional metamorphism. Hydrothermal processes that accompanied the formation of ophicalcites are not disputed; however, they cannot be traced by oxygen isotope geochemistry.

## Introduction

Ever since the pioneering work of Bärtschi (1957), a large amount of data on the stable isotope composition of Alpine carbonate rocks and minerals has been assembled. Isotope data of sedimentary, igneous or metamorphic rocks serve as proxies of past environmental and geochemical conditions, the evolution of the carbon cycle through time, the reconstruction of palaeotemperatures in marine sediments and the identification of the source of magmatic rocks or the composition and origin of fluids acting during diagenesis, and regional and contact metamorphism.

Several early oxygen isotope studies focused on carbonates derived from Jurassic tectono-sedimentary ophiolite breccias, known as ophicalcites. Oxygen isotope data from ophicalcites originally were interpreted as proxies of hydrothermal origin of the carbonate in these rocks (Barberi et al. 1979; Lemoine et al. 1983); however, other authors questioned the hydrothermal signature preserved in bulk oxygen isotope data. They interpreted oxygen isotope trends measured in Alpine ophicalcites and associated pelagic sediments as proxies of Alpine metamorphism (Weissert and Bernoulli 1984; Früh-Green et al. 1990). In a recent study, Coltat et al. (2019) rejected the proposed metamorphic origin of oxygen isotope signatures in Alpine ophicalcites. They use, in agreement with the early studies by Barberi et al. (1979) and Lemoine et al. (1983), oxygen isotope values from Alpine ophicalcites as a proxy of Jurassic oceanic hydrothermal processes.

**Fig. 1:**
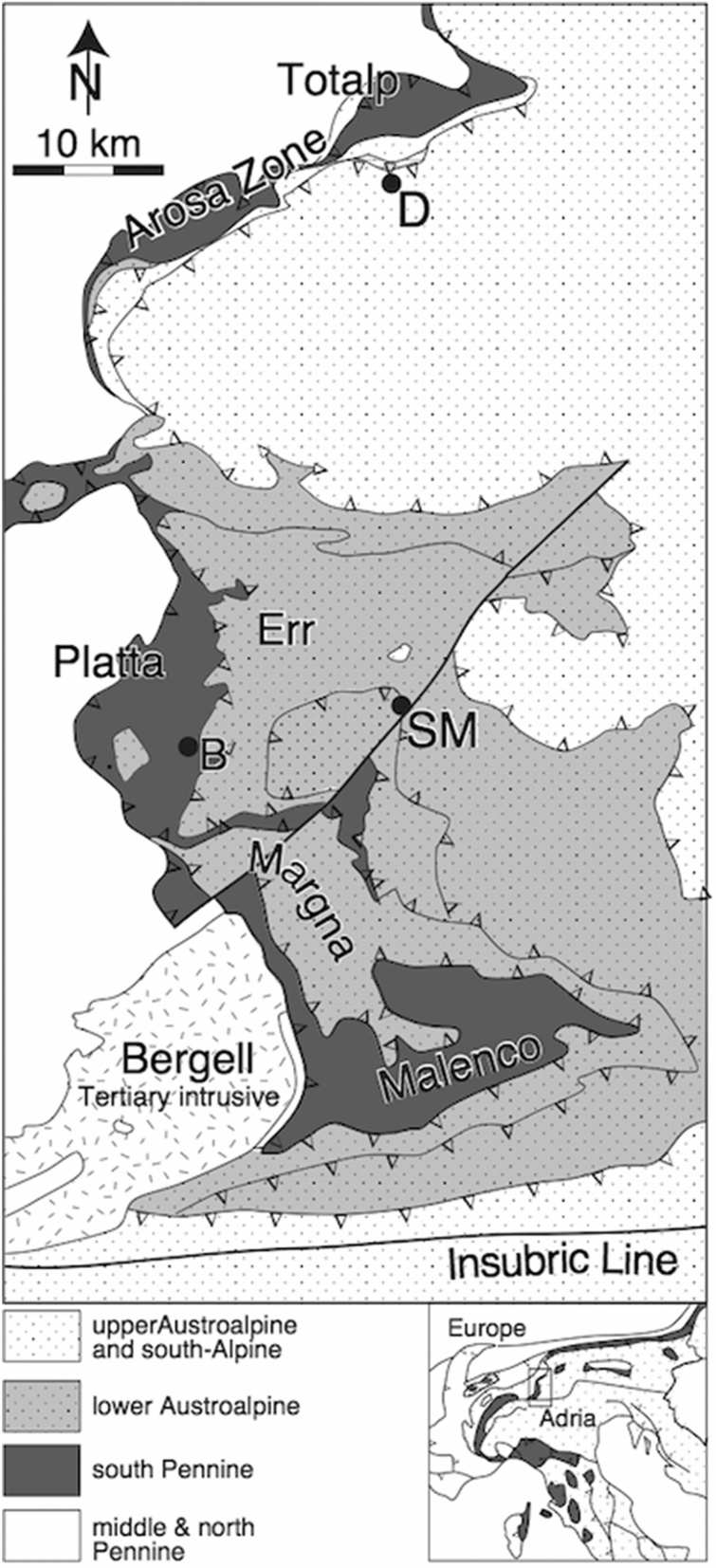
Tectonic units of the Penninic/Austroalpine boundary zone, Eastern Alps, Graubünden, eastern Switzerland. After Spicher (1980)

**Fig. 2:**
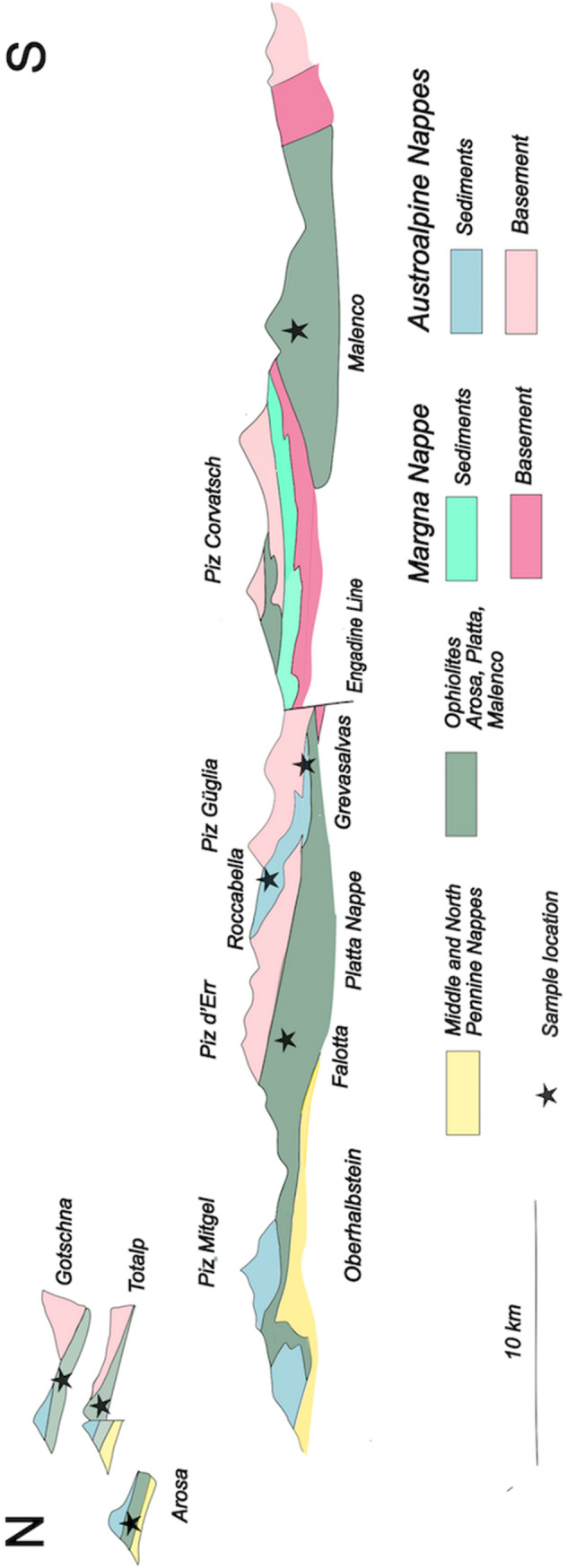
N–S-oriented profile across the Alps of Graubünden with location of the analyzed samples (Fig. 4). Alpine metamorphism is increasing from the upper tectonic units to the lower ones and from the north to south. After Trümpy (1975, modified)

In this short contribution we use earlier published O- and C-isotope data from tectono-sedimentary breccias (ophicalcites) and calcite veins in serpentinites formed along a Jurassic ocean-continent transition in the Alpine Tethys Ocean together with O- and C-isotope data from overlying Jurassic and Cretaceous pelagic sediments (Weissert and Bernoulli 1984; Früh-Green et al. 1990) and discuss them in their regional geological and tectonic context (Figs. 1, 2). We resume a long-lasting discussion on the significance of oxygen isotope values in both tectono-sedimentary breccias and pelagic limestones along the Penninic/Austroalpine boundary zone of the Swiss Alps. In particular, we try to answer the question whether the oxygen isotope composition in ophicalcites is a signal related to Jurassic oceanic hydrothermal processes (Coltat et al. 2019) or is, for its larger part, an Alpine metamorphic signal as postulated by us in earlier papers (Weissert and Bernoulli 1984; Früh-Green et al. 1990).

## Geological setting

The Penninic/Austroalpine boundary zone in the Alps of Graubünden (eastern Switzerland) preserves the remnants of a Jurassic ocean-continent transition (e.g. Froitzheim and Manatschal 1996; Manatschal and Bernoulli 1998, 1999). Alpine thrusting led to the formation of a nappe edifice (Figs. 1, 2) in which the distal continental margin units are represented by the Lower Austroalpine Err and Julier (= Bernina) nappes; the exhumed mantle rocks, by the ophiolite nappes, i.e. the Malenco Unit, the Platta and Totalp nappes with their sedimentary cover (Weissert and Bernoulli 1985). Smaller imbricates of these units occur in the so-called Arosa Zona, a complex assemblage of imbricates of continental margin and ophiolitic units. The Margna Nappe (Fig. 2) occupies an intermediate position sandwiched between the ophiolite units: the present position of this continental margin fragment might reflect its origin as an extensional allochthon (Froitzheim and Manatschal 1996). Subduction and formation of west-vergent thrust nappes began during the Late Cretaceous and was followed by extension soon after; during the Cenozoic, the Cretaceous/Paleocene nappe edifice was thrust to the north over the middle and lower Penninic units (Fig. 2; Froitzheim et al. 1994). The Alpine metamorphic zonation appears to be affected by later, post-nappe thrusting and folding (Ferrero Mählmann 1995), and subsequent extension between 45 and 30 Ma (Nievergelt et al.^1996^); however, by and large, Alpine metamorphism traced in the ophiolitic nappes is of Late Cretaceous to Paleocene age (Handy et al. 1996; Ferreiro Mählmann and Giger 2012; Picazo et al. 2019) and increases along a north–south gradient in southeast Switzerland, exposing deeper units of the Cretaceous–Paleocene nappe edifice (Fig. 2). Oterdoom (1978) studied tremolite- and diopside-bearing assemblages in ultramafic rocks between Davos in the north and the Engadine Valley in the south. He arrived at metamorphic temperatures starting at 200 °C near Davos (Totalp nappe, prehnite-pumpellyite [sub-greenschist] facies) and reaching up to 400 °C in the Engadine Valley (high greenschist facies). In the Platta nappe (sample location Falotta, Fig. 2, see also Coltat et al. 2019), Alpine metamorphism reached pumpellyite to lower greenschist facies.

**Fig. 3:**
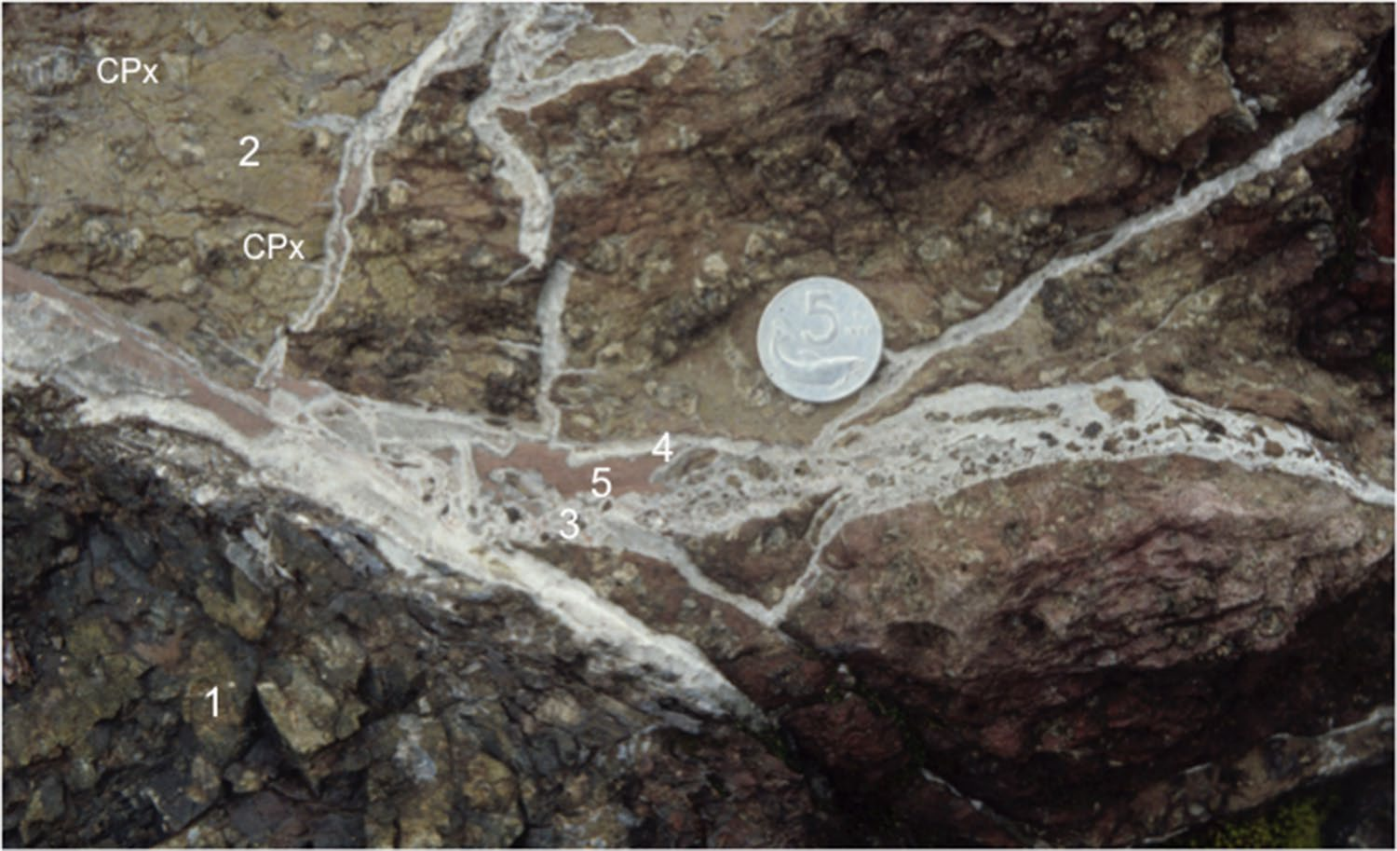
Typical fabric of Alpine ophicalcite. (1) Brecciated serpentinite; (2) In situ-replacement of serpentine minerals by calcite is illustrated by clinopyroxene crystals (CPx) floating in a microsparitic limestone matrix, reflecting the original texture of the fractured and brecciated serpentinite exhumed to the sea floor. (3) Geopetal sediment of small serpentinite fragments in a fracture, cemented by sparry calcite; (4) Sparry calcite lining the void created by the fracture; (5) Late infill of red marine and/or diagenetic sediment, now microsparite. Totalp serpentinite. The Totalp nappe is a lateral equivalent of the Platta nappe (Falotta), which, however, in a more external position underwent only weaker Alpine metamorphism. Totalp, Davos, Switzerland. Coin is 20 mm across. After Bernoulli and Jenkyns (2009)

In spite of Alpine thrusting and imbrication, the original palinspastic sequence of the different units can be reconstructed with confidence from the distal continental margin of the Err nappe to the exhumed mantle rocks of the Platta nappe. The serpentinised mantle rocks of what is now the Platta thrust nappe were exhumed during Jurassic rifting along a system of low-angle detachments and emplaced at the seafloor before being covered by the submarine volcanics and sediments. The intrusion of gabbros into the mantle rocks and the emplacement of the volcanics at the seafloor overlapped in time with the extensional movements as shown by syn-magmatic deformation of the intrusive rocks and their tectonic exhumation at the seafloor (Desmurs et al. 2001). High-temperature deformation and subsequent hydration of the mantle and intrusive rocks was followed by low-temperature deformation, both well documented in the Falotta area of the Platta nappe (Desmurs et al. 2001). The top of the serpentinites is typically formed by tectono-sedimentary breccias, described as ophicalcites which record deformation under decreasing temperatures during ascent of the mantle rocks (Desmurs et al. 2001), hydrothermal activity (Früh-Green et al. 1990), and their final emplacement at the sea floor. The ophicalcites display different phases of carbonation and mineral formation, namely in situ-replacement of serpentine minerals by calcite (Desmurs et al. 2001: Fig. 6c), hydrothermal formation of andradite and tremolite (Früh-Green et al. 1990), cementation by drusy calcite and infill of geopetal marine and/or diagenetic sediment (Fig. 3, Bernoulli and Weissert 1985).

## The oxygen isotope composition of ophicalcites and associated sediments

For the ophicalcites underlying the metabasalts at Falotta, Coltat et al. (2019) claim a Jurassic high-temperature origin. The Jurassic origin of the ophicalcites is beyond doubt as they are overlain stratigraphically by Middle to Upper Jurassic radiolarites that also include re-sediments yielding clasts of ophicalcite (Desmurs et al. 2001: Fig. 6d). Coltat et al. (2019) measured oxygen and carbon isotope compositions of calcite in veins and fractures and found very uniform O- and C-isotope patterns. All C-isotope values are marine values (0.5–2*‰* δ^13^C) corresponding to the values measured in other ophicalcites of the Alpine–Mediterranean orogenic belt (e.g. Barberi et al. 1979; Weissert and Bernoulli 1984; Früh-Green et al. 1990; Pozzorini and Früh-Green 1996; Schwarzenbach et al. 2013, Clerc et al. 2014). This is not surprising because the carbon isotope signature in carbonate of the ophicalcite is rock-dominated, and the carbonate in the ophicalcite is ultimately derived from seawater circulating in the mantle rocks driving serpentinisation. Weissert and Bernoulli (1984) measured like Coltat et al. (2019) exclusively marine carbon isotope values in ophicalcites and, also, in associated Jurassic-Cretaceous pelagic rocks along the described metamorphic gradient.

**Fig. 4:**
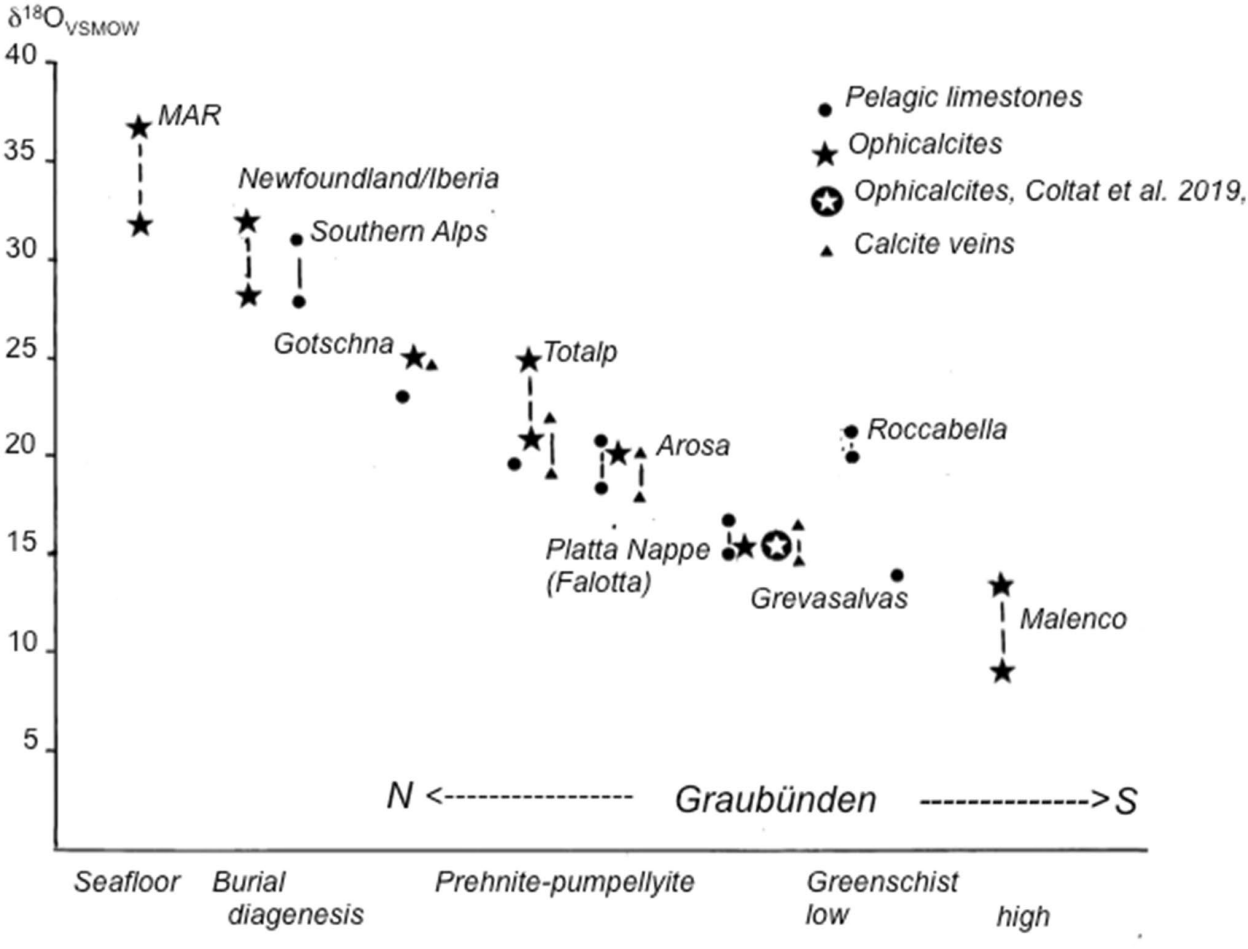
Correlation between O-isotope composition of ophicalcites and oceanic sediments, and metamorphic grade along a transect of the Penninic/Australpine boundary zone, Graubünden. Data from Weissert and Bernoulli (1984), Früh-Green et al. (1990), Pozzorini and Früh-Green (1996). Compilation of values from the Mid Atlantic Ridge (MAR) and from the Iberian and Newfoundlad Margins (IB, NFL) after Picazo et al. (2020)

The interpretation of the O-isotope values has been discussed more controversially (Fig. 4). Whereas Coltat et al. (2019) interpreted their data as a proxy for a Jurassic hydrothermal origin of the calcites in the ophicalcites, we interpreted these values in our earlier papers as an Alpine-metamorphic signal (Weissert and Bernoulli, 1984; Früh-Green et al. 1990). To test the hypothesis that not hydrothermal activity but Alpine metamorphism caused the resetting of the O-isotope composition in ophicalcite, Weissert and Bernoulli (1984) and Früh-Green et al. (1990) measured the O-isotope composition of the overlying pelagic limestones (Calpionella Limestone [Maiolica] and Palombini Formations, uppermost Jurassic and Lower Cretaceous) along a north–south running profile. Indeed, if the O-isotopes in the ophicalcites were an original signature reflecting primarily hydrothermal activity and/or a thermal imprint caused by the overlying basalts, we would expect the O-isotope signatures of overlying oceanic sediments, radiolarites (Middle to Upper Jurassic) and pelagic limestones (Calpionella Limestone and Palombini Formation) to be different from those of the ophicalcites, i.e. showing low-temperature values corresponding to Jurassic–early Cretaceous seawater.

Our profile across eastern Switzerland between Davos and the Engadine includes tectonic units of different Alpine-metamorphic grade (Oterdoom 1978; Bousquet et al. 2012). It definitely displays a parallel trend in O-isotope composition of calcite in ophicalcites and of bulk carbonate of overlying Jurassic-Cretaceous oceanic sediments. Pelagic bulk carbonate values near Davos are up to 5*‰* lower than in their equivalent non-metamorphic rocks in the Southern Alps, known as Maiolica Limestones (e.g. Weissert et al. 1985). An oxygen isotope gradient can be drawn starting from the non-metamorphic Southern Alps to prehnite–pumpelleyite facies in more external units (Totalp–Davos, Arosa) and finally ending in higher greenschist facies in the south-Penninic and Austroalpine nappes in the Engadine. In the Southern Alps (shallow burial diagenesis, < 100 °C), δ^18^O_V-PDB_ values of −3 to 0*‰*, corresponding to δ^18^O_V-SMOW_ values of 28–31*‰* were measured in Lower Cretaceous pelagic limestones (Maiolica Lombarda Formation); at Totalp (Davos, Totalp Nappe) and Arosa (Arosa Zone) (prehnite–pumpellyite facies) values of 19–24*‰* δ^18^O_V-SMOW_ (δ^18^O_V-PDB_ = − 7 to − 10*‰*) in the ophicalcites and the overlying sediments. At Falotta, all values measured by Coltat et al. (2019) fall around + 16*‰* δ^18^O_V-SMOW_ (− 14.5*‰* δ^18^O_V-PDB_) (Fig. 4). These values are in perfect agreement with oxygen isotope compositions measured by Weissert and Bernoulli (1984) and by Früh-Green et al. (1990) at the same location in calcite veins cutting serpentinites and in ophicalcites. δ^18^O_V-PDB_ values in pelagic sediments (Palombini Formation, Lower Cretaceous) of the same area are around − 10.5*‰* δ^18^O_V-PDB_ (20*‰* δ^18^O_V-SMOW_) with lowest values near − 16*‰* δ^18^O_V-PDB_ (14*‰* δ^18^O_V-SMOW_) along lithological boundaries testifying to *post-Jurassic* isotope fractionation during recrystallization of calcite (Eppel and Abart 1997). Further to the south, near the contact aureole of the Oligocene Bregaglia (Bergell) intrusion (Fig. 1), the O-isotope values in Jurassic-age ophicalcites of the Malenco complex range from 8 to 13*‰* δ^18^O_V-SMOW_ (− 22 to − 17*‰* δ^18^O_V-PDB_) whereby the range decreases with distance from the intrusive contact to 11–12*‰* δ^18^O_VSMOW_ (− 19 to − 18*‰* δ^18^O_V-PDB_; Pozzorini and Früh-Green 1996).

The oxygen isotope compositions in both ophicalcites and pelagic limestones show exactly the same trend of decreasing oxygen isotope values starting from Davos in the north and ending south of the Engadine valley. The same trend in O-isotope values has been recognized long ago in ophicalcites and overlying oceanic sediments in the Apennines where the O-isotope values become more negative from the more metamorphic Ligurian units in the north to their less metamorphic counterparts in the south (Bonatti et al. 1974; Barberi et al. 1979). The ultramafic carbonate breccias from the Mid-Atlantic Ridge vary from 2.5 to 5*‰* δ^18^O_V-PDB_ or 33.5–36*‰* δ^18^O_V-SMOW_ (Bonatti et al. 1974); fracture-filling calcites from the Iberian distal margin from − 0.8 to 1*‰* δ^18^O_V-PDB_ or around 30 *‰* δ^18^O_V-SMOW_ (Millikan and Morgan 1996), and oxygen isotope values of calcites from Cretaceous ophicalcites of the Newfoundland Margin cluster in a relatively restricted range of 30.8 to 32.6*‰* δ^18^O_V-SMOW_ for replacive calcite grains and at 28 *‰* δ^18^O_V-SMOW_ in calcite veins (Picazo et al. 2020). Indeed, all cited examples from the Atlantic Ocean formed near or at the sea floor and at low temperatures (< 20 °C), even where syn- and post-rift magmatism occurs as in the Platta unit. By contrast, the uniform low O-isotope values of the ophicalcites at Falotta argue for a late, metamorphic origin of the O-isotope composition. Indeed, we would expect that the different generations of calcite observed in ophicalcite would display more widely varying O-isotope signatures if they formed at different depth below the Jurassic seafloor, but they do not; all lie slightly above − 14*‰* δ^18^O_V-PDB_ (16.5*‰* δ^18^O_V-SMOW_), in the range that also the overlying oceanic sediments display. No low-temperature ophicalcites have been found yet in the Alps, suggesting that their original low-temperature signatures have been obliterated by Alpine metamorphism.

**Fig. 5:**
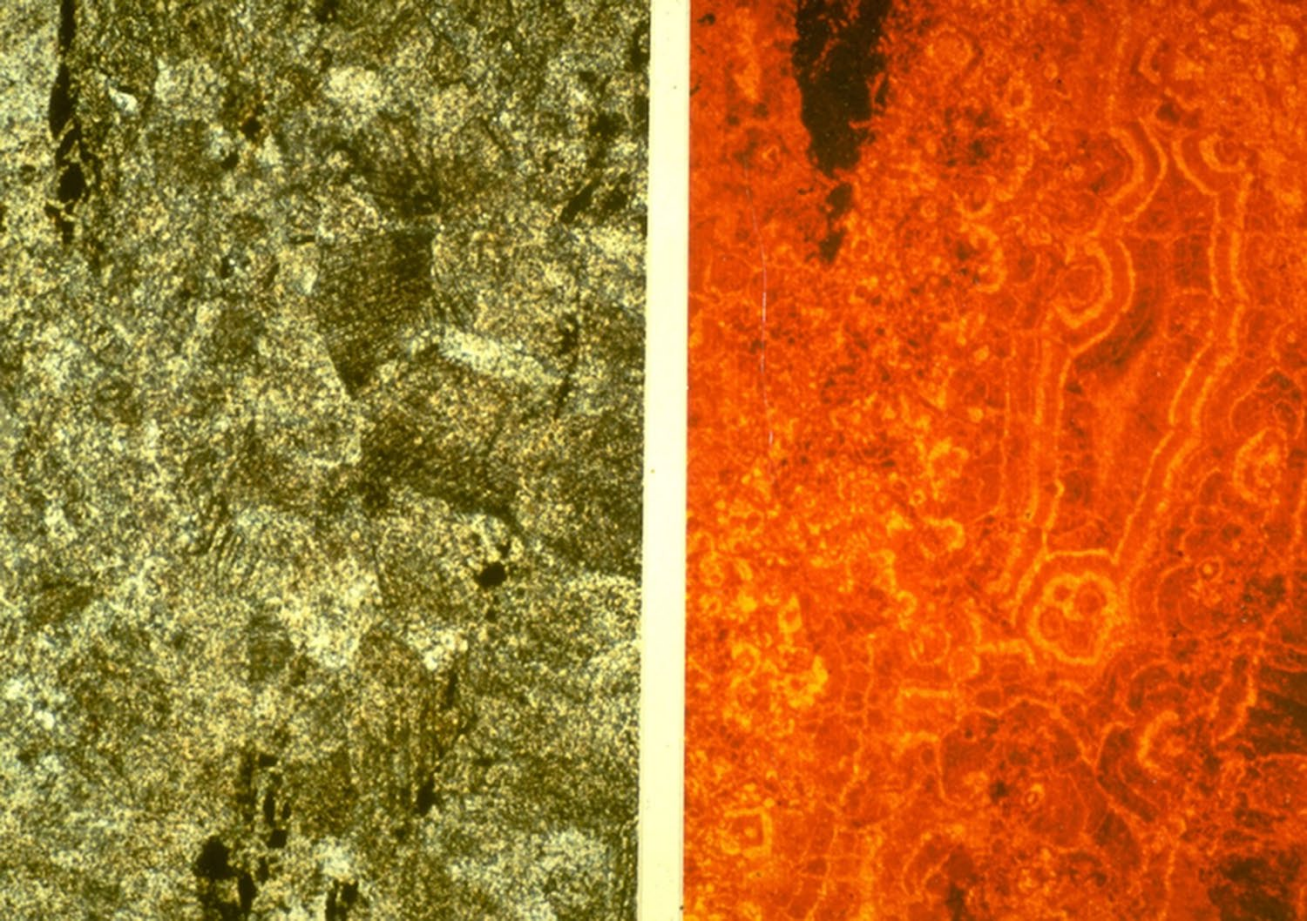
Carbonate texture in ophicalcite. A recrystallized calcite fabric (left) interpreted as related to Alpine overprint, hides a palimpsest of undeformed bothryoidal calcite-cement texture visible under cathode-luminescence (right) showing alternating laminae of different hues of orange color related to the assimilation of Mn^+2^ testifying to reduced fluids, Totalp, Davos, Switzerland. Field of view (vertical) is 3 mm. After Früh-Green et al. (1990)

When were the isotope compositions in the pelagic sediments and the ophicalcites homogenized? Subduction of the “South Penninic” Ocean with its ophiolites started in the Late Cretaceous. Pelagic ooze of latest Jurassic and Early Cretaceous age entered the accretionary wedge as a fluid-rich stiff carbonate ooze to porous chalk. Such pelagic coccolith oozes of Early Cretaceous age of the same facies as the Calpionella Limestone (Maiolica facies) occur still today in the western Central Atlantic where they have been drilled by the Deep Sea Drilling Project (Bernoulli 1972). Deep Sea Drilling shows at many places that burial diagenesis altered pelagic carbonate ooze below the first hundred meters to a stiff ooze. With burial depths of a few hundred meters, ooze is replaced by porous chalk accompanied by a decrease in porosity and in the δ^18^O_V-PDB_ values from + 1 to − 3 *‰* as described from the Central Pacific by Matter et al. (1975). Obviously, fluid-rich young sediments were lithified further and recrystallized during accretion of the wedge accompanied by fluid expulsion during subduction. Isotopic resetting was not limited to pelagic sediments but equally involved the underlying ophicalcites as underlined by Alpine metamorphic recrystallisation textures (Fig. 5). Eppel and Abart (1997) analyzed C- and O-patterns in the Platta Nappe along tectonic contacts between serpentinites and Lower Cretaceous Palombini sediments: 20 m above a tectonic contact, Palombini sediments showed the expected O-isotope values resulting from subduction-related isotope resetting. Close to the thrust contact, a shift in both O- and C-isotope values of several permil records re-equilibration of the affected rocks by fluids channelized along Alpine tectonic contacts. None of the samples used in our studies (Figs. 2, 4) were taken near thrust contacts. The fact that local isotopic exchange occurred along Alpine tectonic contacts corroborates our view that oxygen isotope compositions in carbonate rocks record Alpine metamorphic processes and not pre-Alpine oceanic events.

Interestingly, Jurassic carbonated fault rocks and ophicalcites of the Bracco-Val Graveglia Unit of the Liguride nappes in the Northern Apennines showed δ^18^O values of 19.8 to 20*‰* δ ^18^O_V-SMOW_ for the fault rocks (Alt et al. 2018), and between 13 and 20*‰* δ ^18^O_V-SMOW for_ the ophicalcites (Schwarzenbach et al. 2013). These values are in the range of those of ophicalcites of the Platta nappe and the Arosa Zone in Graubünden. In fact, Lucchetti et al. (1990) and Leoni et al. (1996) established, based on mineral assemblages in the ophiolites and illite/chlorite crystallinity in the sediments overlying them, prehnite-pumpellyite facies and low-grade metamorphic (anchizone) conditions for the Bracco-Val Graveglia Unit. Also, in these cases Alpine isotopic exchange is probable.

## Conclusion

We do not doubt the importance of hydrothermal activity, accompanying serpentinisation and fracturing for the carbonation of the ophicalcites that we documented in an earlier paper (Früh-Green et al. 1990). Hydrothermal activity is testified to in the mineralogy, in cathode luminescence analysis of calcite veins (Fig. 5) but not in the measured oxygen isotope values of ophicalcites that follow a regional metamorphic trend (Früh-Green et al. 1990). All the oxygen isotope values measured in Alpine ophicarbonates reflect isotopic resetting during Alpine orogeny, related to fluid-rock interaction during regional metamorphism and show that pervasive metamorphic fluids, derived from metamorphic reactions caused local or regional widespread homogenization of the O-isotope compositions. C-isotope values were not affected because the C-isotope system was rock-dominated during the Alpine metamorphic event.
